# Sensor and Sensorless Technology with Renewable Energy and Flexible Load Participation in Active Distribution Network

**DOI:** 10.3390/s25154815

**Published:** 2025-08-05

**Authors:** Ning Li, Jie Yan, Su Su, Jakub Jurasz, Rongsheng Chen

**Affiliations:** 1 School of Electrical Engineering, Xi’an University of Technology, Xi’an 710048, China; 2 School of New Energy, North China Electric Power University, Beijing 102206, China; 3 School of Electrical Engineering, Beijing Jiaotong University, Beijing 100044, China; 4 Faculty of Environmental Engineering, Wroclaw University of Science and Technology, 50-377 Wroclaw, Poland; 5 School of Traffic and Transportation, Beijing Jiaotong University, Beijing 100044, China

## 1. Introduction

With the rapid growth of active distribution networks, the demand for intelligent and flexible operation has increased significantly. To meet this need, integrated sensing and sensorless technologies have gained attention for their ability to efficiently handle renewable integration, equipment status, faults, and dispatch strategies while improving system visibility and control through multi-source data fusion. Recent studies show these technologies are widely used in fault detection, recovery, dispatch optimization, image processing, and data modeling in transmission lines and renewable energy facilities.

## 2. Overview of Contributions

This Special Issue aims to provide cutting-edge solutions to the challenges of fault state identification in active distribution networks and to offer valuable references for future theoretical research and practical deployment in the field. It includes ten high-quality papers covering several key issues regarding active distribution networks, primarily focusing on the following three areas: equipment condition detection and fault identification [1,2,3]; dispatch optimization and fault restoration [4,5,6,7]; image processing and data-driven intelligent modeling [8,9,10].

To detect faults in key power components, Wang et al. [1] integrated fuzzy Petri nets, backpropagation neural networks, and Dempster–Shafer (DS) theory for improved diagnosis in direct current (DC) converter stations. Wang et al. [2] developed You Only Look Once–Small Size–Large (YOLO-SS-Large) for efficient small-target detection in substations. Wang et al. [3] addressed limited unmanned aerial vehicle (UAV) image data by combining You Only Look Once version 5 (YOLOv5) with augmentation for insulator anomaly detection.

In active distribution systems with multi-source coordination and diverse stakeholders, several strategies have been proposed for efficient fault recovery and coordinated operation. Cao et al. [4] introduced a distributed virtual inertia control scheme using neighbor communication to enhance frequency response under conditions of high renewable penetration. Dang et al. [5] proposed a graph-based, multi-sensor restoration method for complex topologies, improving recovery efficiency. Wu et al. [6] developed a two-layer game-theoretic optimization model for multi-microgrids with shared energy storage, ensuring fair benefit distribution via an improved Shapley value. Guo et al. [7] designed a wide-area thyristor-controlled series capacitor (TCSC)-based active distribution network (ADN) architecture and a deep reinforcement learning-driven control strategy to enhance renewable integration and operational flexibility in complex terrains.

To enhance image processing and prediction accuracy, this Special Issue presents three studies. Luan et al. [8] employed federated learning for insulator fault detection, ensuring data privacy without sacrificing performance. Zhang et al. [9] developed a conditional generative adversarial network–convolutional neural network–long short-term memory (CGAN-CNN-LSTM) hybrid model using Light Detection and Ranging data for accurate ultra-short-term wind power forecasting. Ma et al. [10] proposed a probabilistic model with the expectation–maximization algorithm to classify wind turbine operating states, improving the reliability of supervisory control and data acquisition-based analytics.

## 3. Conclusions

In summary, although this Special Issue proposes solutions to various challenges related to equipment fault identification and recovery in active distribution networks, the application of sensing and sensorless technologies under the coordinated participation of renewable energy and flexible loads still faces significant challenges. We hope that this Special Issue can provide a solid theoretical foundation and a practical point of reference for building the next generation of distribution systems with capabilities in perception, autonomous control, and intelligent decision-making.
